# A Text-book of the Theory and Practice of Medicine

**Published:** 1894-07

**Authors:** 


A TEXT-BOOK OF THE THEORY AND PRACTICE OF MEDICINE. By American Teachers. Edited by William Pepper, M.D., LL.D., Provost and Professor of the Theory and Practice of Medicine and of Clinical Medicine in the University of Pennsylvania. In two volumes. Illustrated. Vol. II. Philadelphia: W. B. Saunders, 925 Walnut street. 1894.

The first volume of this text-book was reviewed in this JOURNAL about one year ago. It was highly commented upon by the medical reviewers, and was well received by the profession. The second volume compares favorably in every way with the first. The subject-matter of this great work has been so divided out that each subject is discussed by an expert of special authority. Drs. H. C. Wood, J. C. Wilson, J. T. Whittaker, W. H. Welch, W. G. Thompson, William Pepper, William Olsler, H. M. Lyman, E. G. Janeway, J. W. Holland, R. H. Fitz, Francis Delafield, J. S. Billings are the authors, and a more able set of teachers cannot be found. The masterly manner in which each subiect is handled does great credit to even these brilliant writers. As Dr. Pepper says, it represents truly the best teaching of the science and art of medicine at the present time in this country. V. H. T.

---

MR. SAUNDERS is pleased to announce, as in *active preparation*, his "New Aid Series of Manuals for Students and Practitioners."

As publisher of the "*Standard Series of Question Compends*," together with an intimate relation with leading members of the medical profession, Mr. Saunders has been enabled to study, progressively, the essential *desideratum* in practical "self-helps" for students and physicians. This study has manifested that, while the published "Question Compends" earn the highest appreciation of students, whom they serve in reviewing their studies preparatory to examination, there is special need of thoroughly reliable hand-books on the leading branches of medicine and surgery, each subject being compactly and authoritatively written, and exhaustive in detail, without the introduction of *cases* and foreign subject-matter which so largely expand ordinary text-books. The *Saunders Aid Series* will not merely be condensations from present literature, but will be ably written by well-known authors and practitioners, most of them being teachers in representative American colleges. This *new series*, therefore, will form an admirable collection of advanced lectures, which will be invaluable aids to students in reading and in comprehending the contents of "recommended" works.

Each Manual, comprising about 250 pages (5½x8 inches), will further be distinguished by the beauty of the *new* type; by the quality of the paper and printing; by the copious use of illustrations; by the attractive binding in cloth; and by the extremely low price, which will uniformly be $1.25 per volume.

